# Risk of Transmission of COVID-19 from the Mother to the Foetus: A Systematic Review

**DOI:** 10.34763/jmotherandchild.20242801.d-24-00032

**Published:** 2024-11-20

**Authors:** Ermioni Palaska, Eleni Golia, Evgenia Zacharogianni, Anastasia Bothou, Maria Tziriridou-Chatzopoulou, Maria Dagla, Evangelia Antoniou, Eirini Orovou

**Affiliations:** Department of Midwifery, University of West Attica, Egaleo, Greece; Department of Midwifery, University of Western Macedonia, Ptolemaida, Greece

**Keywords:** vertical transmission, Coronavirus disease, COVID-19, SARS-CoV-2, pregnancy, obstetric outcomes

## Abstract

**Introduction:**

People’s lives have been impacted in every way by the COVID-19 pandemic and it had a variety of effects on pregnancy and childbirth, including decreased access to healthcare providers who can attend to the needs of expectant mothers and their foetuses. These effects can be attributed to the infection’s effects on the mother and foetus.

**Aim:**

The aim of this research was to investigate the probability of vertical transmission of COVID-19 from the pregnant mother to the foetus.

**Methods:**

A comprehensive systematic search was conducted on the PubMed, SCOPUS, and Web of Science databases to identify original research articles published from 2019 to 2021. The search aimed to locate cohort studies, case series, and reports focusing on pregnant individuals with COVID-19, specifically those containing information on COVID-19 testing for foetuses or newborns.

**Results:**

In this systematic review, studies showed that the possibility of vertical transmission from a COVID-19-infected mother to the foetus or neonate is rare.

**Conclusion:**

With regards to the theoretical framework proposed regarding the vertical transmission of COVID-19 from the pregnant woman to the foetus or neonate, there exists a potential risk of transmission. Nevertheless, documented instances of confirmed vertical transmission are limited and inadequately documented in the available literature.

## 1. Introduction

The outbreak of coronavirus disease (COVID-19), which is caused by the severe acute respiratory syndrome coronavirus 2 (SARS-CoV-2), was initially reported as an epidemic in China (specifically, Wuhan, Hubei Province) in December 2019 [1]. Merely three months following the initial outbreak, the World Health Organization (WHO) characterised the spread of the disease as a worldwide pandemic [2], signalling an urgent public health emergency of international significance attributed to its high transmissibility and associated morbidity and mortality rates. Until then, 118,000 cases had been identified in 114 countries, 4,291 people had lost their lives, and thousands of others were fighting to save their lives in hospitals [2].

Since then, people’s lives have been affected, and the fear of the unknown virus has also burdened their psychosocial health [3] and had a variety of effects on pregnancy and childbirth. These effects can be attributed to the infection’s effects on the mother and foetus (4–5).

The virus usually spreads through droplets among individuals who are in close contact. Many people are asymptomatic or have mild symptoms. However, for some elderly individuals and those with certain medical conditions, such as chronic lung diseases, neurological diseases, heart and kidney diseases, diabetes and obesity, COVID-19 may require additional hospital care or may lead to death. In addition, immunocompromised people and pregnant women, as well as disabled patients, are at increased risk of illness and the use of additional medical care [6]. It is also important to mention that heart diseases and urinary system disorders tend to have a greater negative impact on COVID-19 and are more likely to lead to death [7]. Existing evidence suggests that pregnancy does not increase susceptibility to infection by the virus but significantly worsens the clinical course of COVID-19 (e.g., increased admission to the intensive care unit [ICU], need for respiratory support, and mechanical ventilation, and ultimately higher chances of death) compared to non-pregnant women with similar characteristics who are symptomatic [8]. However, during the pandemic, it was observed that pregnant women discontinued their regular prenatal visits, and several pregnant women were admitted to the emergency department with urgent conditions requiring acute intervention. This led to an increase in the number of cases diagnosed with Small for Gestational Age (SGA) and hypoxic-ischemic encephalopathy in newborns [9].

However, one of the most important outcomes of a confirmed COVID-19 infection during pregnancy is the potential transmission of the infection to the foetus or newborn. The term “vertical transmission” refers to the transmission of a pathogen from the mother to the foetus during both prenatal and postnatal stages. It can occur through the blood or infected particles in the placenta during pregnancy, during delivery through the birth canal, and through breastfeeding [10,11].

There are several reasons why the vertical transmission of COVID-19 is a significant concern, one of which is the known tissue tropism of COVID-19, but mainly there is mothers’ anxiety and the incomplete data on the virus’ behaviour. According to the data, many mothers who were infected with the SARS-CoV-2 chose or underwent caesarean delivery to reduce the risk of neonatal infection. However, there are no reports of neonatal infection through vaginal delivery, as all reported cases of vaginal birth showed negative results in the reverse transcription-polymerase chain reaction (RT-PCR), indicating that the newborn was not infected during delivery [13].

Regarding the transmission of the virus, the primary receptor responsible for facilitating the entry of COVID-19 into a cell is the angiotensin converting enzyme 2 (ACE2) receptor. ACE2 expression is noted in the placenta [13] and is localised within the syncytiotrophoblast, cytotrophoblast, endothelium, and vascular smooth muscle [14] of primary and secondary veins, as well as in the vagina. ACE2 expression is detected in various tissues indirectly connected to the development of pregnancy. Furthermore, recent case studies suggest that COVID-19 can potentially infect the placenta, as evidenced by the presence of viral RNA and SARS-CoV-2 protein in the placenta, along with the identification of viral particles within the syncytiotrophoblast [15,16,17]. In cases of congenital viral infection, the placenta can exhibit various pathological changes. These include hematogenous placentitis, which is characterised by inflammation of the placental villi (villitis). The severity of this condition can vary, and in more severe cases, it can lead to granulomatous myometritis (inflammation of the uterine muscles) and microabscesses, which are small accumulations of pus. In rare cases, viral particles can be detected in the placenta. Some viruses can cross the placenta and cause additional damage to the villi, which are often more stromal than inflammatory. Such damages include delayed maturation of the villi and cellular hyperplasia of Hofbauer cells [18]. A characteristic example is congenital Zika syndrome, where stromal lesions are more prominent than inflammatory ones [19].

## 2. Aim

Based on the above, a systematic review was conducted with the aim of investigating the possibility of vertical transmission of COVID-19 infection from mother to foetus.

### Patients and Methods

This study complied with the guidelines of the PRISMA statement. “A systematic search was conducted on the PubMed, SCOPUS, and Web of Science databases to identify original research studies published between 2019 and 2021 (the specific period was chosen because it concerns the first waves of the pandemic with the most virulent mutations) to identify cohort studies and case series, focusing on pregnant women with COVID-19 that contained details on foetal or neonatal COVID-19 testing, eligible studies were identified through a search using a mix of controlled vocabulary and full-text terms.

**Figure 1.**
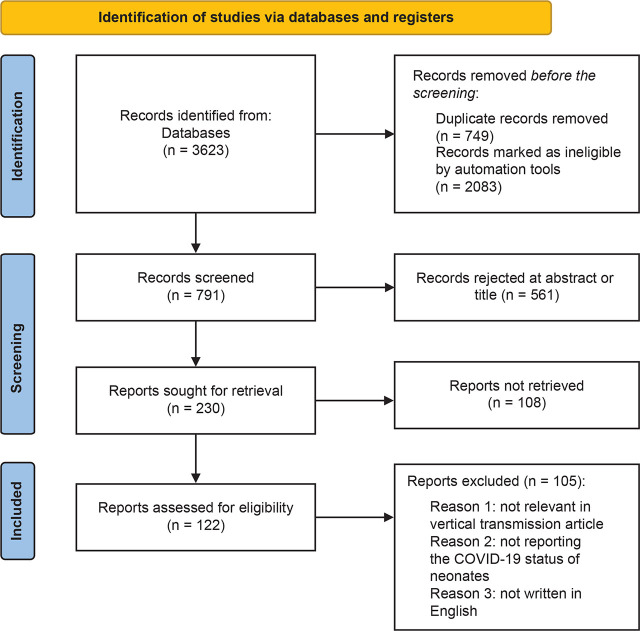
Results of studies based on the PRISMA method.

The search terms were (SARS-CoV-2[MeSH Terms]) OR (COVID-19[MeSH Terms]) OR (coronavirus[MeSH Terms])) OR (Corona viridae[MeSH Terms])) AND (pregnancy[MeSH Terms])) OR (fetus[MeSH Terms])) OR (infant[MeSH Terms])) OR (mother-to-child transmission[MeSH Terms])) OR (mother-to infant transmission[MeSH Terms])) OR (mother-to-fetus transmission[MeSH Terms])) OR (vertical transmission[MeSH Terms]) Filters: Full text, Observational Study, English, Humans, from 2019–2021. This systematic review was not submitted for registration in PROSPERO. References from all databases were imported into a Mendeley library. Figure 1 shows the PRISMA flowchart concerning the selection of the studies.

The study population comprised pregnant or post-partum women who had the experience of a COVID-19 infection.

So, the exposure was defined as a COVID-19 infection during pregnancy and outcomes were incidence of vertical transmission. The inclusion criteria were as follows: the analysis was performed in pregnant women affected by COVID-19 and the studies were original papers written in English. Therefore, articles not published in English, not assessing COVID-19 infection on pregnant women as well as meta-analysis systematic reviews, letters to editor, and reviews were all excluded.

The search strategy followed retrieved 3623 studies. After removing 749 duplicates and 2083 titles involving studies that were published before 2019, as well as review articles, 791 titles and abstracts were collected. Of the 791 titles, 561 were excluded as they were non-relevant topics. Of the remaining titles, 108 were excluded as it was not possible to retrieve the full articles. Finally, a further 105 studies were removed as during the review of the articles it was judged that they did not relate to vertical transmission, deficiencies were found in the reporting of positive or negative COVID-19 tests in newborns as well as some studies were not in the English language. The final titles that were included in the systematic review were 17.

## 3. Results

The present systematic review encompassed 17 articles “Table 1” with multiple methodologies, including 11 retrospective studies, three cohorts and four case studies. In more details, in a retrospective study conducted by H. Chen et al. [20], the study reported on the condition of nine pregnant women diagnosed with COVID-19. The presence of SARSCoV-2 was investigated in six out of the nine cases using amniotic fluid, breast milk, cord blood, and neonatal saliva smears. The results revealed that all tests returned negative findings, including the six neonatal samples that were tested for SARS-CoV-2 [20]. The results were similar to the study by Chen et al. who reported that no vertical transmission was observed in pregnant COVID-19-positive mothers [21].

**Table 1.** Summary of the 17 included studies in the systematic review

**Author, year (country)**	**Study**	**Population**	**Exposure**	**Main Outcomes**
Chen et al. 2020 (China) [18]	Retrospective Study	Nine pregnant women in the third trimester of pregnancy and their neonates	Positive COVID-19 test	There was no indication of vertical transmission of COVID-19 to the neonates
Chen S. et al. 2020 (China) [19]	Case report	Five pregnant women and their neonates	Positive COVID-19 test	There was no indication of vertical transmission of COVID-19 to the neonates
Zhu et al. 2020 (China) [20]	Retrospective Study	Nine women and ten neonates (one twin pregnancy)	Positive COVID-19 test	There was no indication of vertical transmission of COVID-19 to the neonates
Ferrazzi et al. 2020 (Italy) [23]	Retrospective Study	42 women with COVID-19 and their neonates	Positive COVID-19 test	Among the 42 births, three infants tested positive for COVID-19 post-delivery on days one and three, respectively
Liao et al. 2020 (China) [22]	Retrospective Study	Ten women, positive for COVID-19, and ten neonates	Positive COVID-19 test	There was no indication of vertical transmission of COVID-19 to the neonates
Liu et al. 2020 (China) [23]	Retrospective Study	51 women and 51 neonates	Positive COVID-19 test	There was no indication of vertical transmission of COVID-19 to the neonates
Nie et al. 2020 (China) [26]	Retrospective Study	33 women and 28 neonates	Positive COVID-19 test	Only a neonate was found to have SARS-CoV-2 infection
Pereira et al. 2020 (Spain) [27]	Cohort study	60 women and their neonates	Positive COVID-19 test	There was no indication of vertical transmission of COVID-19 to the neonates
Qiancheng et al 2020 (China) [29]	Retrospective Study	82 women and their neonates	Positive COVID-19 test	There was no indication of vertical transmission of COVID-19 to the neonates
Rosen et al. 2021 (Israel) [30]	Cohort study	55 women and their neonates	Positive COVID-19 test	There was no indication of vertical transmission of COVID-19 to the neonates
Wang et al. 2020 (China) [29]	Case study	A woman and her neonate	Positive COVID-19 test	There was no indication of vertical transmission of COVID-19 to the neonates
Yan et al. 2020 (China) [32]	Retrospective Study	116 women and 86 neonates	Positive COVID-19 test	There was no indication of vertical transmission of COVID-19 to the neonates
Breslin et al. 2020 (USA) [33]	Retrospective Study	43 Women and their neonates	Positive COVID-19 test	There was no indication of vertical transmission of COVID-19 to the neonates
Yang et al. 2020 (China) [32]	Retrospective Study	Seven women and their neonates	Positive COVID-19 test	There was no indication of vertical transmission of COVID-19 to the neonates
Yang et al. 2020 (China) [33]	Retrospective Study	24 women and their neonates	Positive COVID-19 test	There was no indication of vertical transmission of COVID-19 to the neonates
Chen Y. et al. 2020 (China) [34]	Case study	Four women and their neonates	Positive COVID-19 Test	There was no indication of vertical transmission of COVID-19 to the neonates

A study conducted by Zhu et al. (2020) reported on nine pregnant women diagnosed with COVID-19 and their ten infants (including a set of twins) from five hospitals in Hubei province. The women’s ages ranged from 25 to 35 years old, with a symptom onset to delivery interval of one to six days. Chest CT scans of all women revealed patchy pulmonary consolidation with blurred borders, characteristic of viral pneumonia. SARS-CoV-2 testing yielded positive results for all patients except for the mother of the twins [22]. The initial symptoms observed in these women were a fever or cough. The well-being of the infants was evaluated using the Pediatric Critical Illness Score (PCIS), with six newborns scoring below 90. Among the infants, six experienced dyspnea, two presented with fever, and one exhibited a rapid heart rate. Throat swab samples were obtained from nine neonates within one to nine days after birth and tested for SARSCoV-2, with all results returning negative [22]. Furthermore, the study by Ferrazzi et al. [23] included 42 women with COVID-19; 24 gave birth transvaginal. Among the 42 cases, elective caesarean sections were performed in 18 instances, with eight cases not attributed to the COVID-19 infection. Pneumonia was identified in 19 cases, with seven requiring oxygen support and four being admitted to an intensive care unit. Two women diagnosed with COVID-19 breastfed without a mask postpartum, resulting in their infants testing positive for SARS-CoV-2 infection. One newborn also tested positive after vaginal delivery [23].

In a retrospective review conducted by Liao et al., medical records were analysed along with a comparison of vaginal delivery outcomes in ten pregnant women diagnosed with COVID-19 and 53 pregnant women without COVID-19 at Zhongnan Hospital of Wuhan University from January 20 to March 2, 2020. No significant differences were observed in gestational age, postpartum bleeding, neonatal birth weight, and rates of neonatal asphyxia between the two groups. Additionally, neonates born to pregnant women with a clinical diagnosis of COVID-19 tested negative for SARS-CoV-2 infection, ruling out vertical transmission of the virus from mother to foetus/neonate in this study [24]. Similar results were obtained in the study by Liu et al. where none of the 51 neonates developed febrile or respiratory distress during hospitalisation [25].

In a study conducted by Nie et al. [26], 33 pregnant women with COVID-19 and 28 neonates were identified. Among the pregnant women, one (3%) required mechanical ventilation and none were admitted to the intensive care unit (ICU). The distribution of symptoms among pregnant women included 13 (39.4%) with mild symptoms, 19 (57.6%) with moderate symptoms, and one (3%) with severe symptoms. Additionally, one neonate (3.6%) developed acute respiratory distress syndrome (ARDS) and was subsequently admitted to the ICU. The rate of perinatal transmission of SARS-CoV-2 was reported as 3.6% [26].

Pereira et al.’s study involved the diagnosis of COVID-19 in 60 pregnant women [27]. The predominant symptoms observed were fever and cough (75.5%), followed by dyspnea (37.8%). Among the participants, 41 women (68.6%) necessitated hospital admission, with 18 admitted due to disease progression and 23 for delivery purposes. Out of these, 21 women (35%) received treatment with medications such as hydroxychloroquine, antivirals, antibiotics, and tocilizumab. No instances of renal or cardiac failure or maternal fatalities were documented. Throughout the study duration, 18 women (78%) delivered vaginally. All neonates were negative for SARS-CoV-2 and none of them were infected during breastfeeding. The study’s significant discovery was the absence of SARS-CoV-2 in placental tissue, as no viral presence was detected, as highlighted in reference [27]. Out of the 64 pregnant women with COVID-19 in Pierce-Williams et al.’s Research, 20 (31%) had critical disease and 44 (69%) had severe disease [28]. Out of 20 women with critical illness, 15 (75%) delivered prematurely. No stillbirths, neonatal deaths, or instances of vertical transmission were reported [28]. A study by Qiancheng et al. similarly concluded that there was no indication of vertical transmission of COVID-19 in the late stages of pregnancy, even during vaginal delivery [29]. Additionally, the research conducted by Rosen et al. determined that SARS-CoV-2 infection in the early stages of pregnancy did not lead to vertical transmission and led to positive obstetric and neonatal outcomes [30]. In a study conducted by Wang et al., the case of a 28-year-old pregnant woman with a one-week history of febrile illness from SARSCoV-2 was detailed. During the hospitalisation, reduced foetal movement and a lack of variability in foetal heart rate were noted on the third day, leading to an emergency caesarean section. A premature male infant weighing 1830g was born. Placenta, amniotic fluid, umbilical cord blood, gastric fluid, and pharynx swabs from the infant were sampled, with all tests yielding negative results for SARS-CoV-2 by RT-PCR. Three days post-delivery, neonatal swab and stool samples also tested negative by RT-PCR. Subsequent tests conducted seven and nine days after birth confirmed negative RT-PCR results for COVID-19 in both the mother and infant [31].

Based on the study by Yan et al., no association was found between coronavirus infection and the development of severe acute respiratory distress syndrome (ARDS) during pregnancy [32]. Furthermore, there is no increased risk of spontaneous abortion or spontaneous preterm delivery due to a coronavirus infection. The study also suggests that when a coronavirus infection occurs in the third trimester of pregnancy, there is minimal evidence of vertical transmission leading to severe acute respiratory syndrome [32]. In the research conducted by Breslin et al., a cohort of 43 pregnant women was identified as positive for COVID-19. Following their births, all neonates underwent COVID-19 testing through nasopharyngeal SARS-CoV-2 PCR smear. Of these, 15 infants were found negative on the first day of life. Two infants had inconclusive results, but the test results were negative when repeated on. One neonate had an “indeterminate” test result, which was clinically treated as an “assumed negative” diagnosis, as this result may reflect low-level detection [33]. Additionally, Yang P. et al., Yang Hu et al. and Chen et al. showed that there was no indication of vertical transmission of COVID-19 to the neonates [34,35,36].

## 4. Discussion

The potential for vertical transmission of SARS-CoV-2 from an infected mother to her foetus or newborn has been a subject of debate. Previous systematic reviews have suggested that there is no conclusive evidence of vertical transmission. Various studies have investigated the impact of COVID-19 on pregnant women, including their clinical outcomes [27,28,35). Although these studies provide valuable information on disease progression and its impact on maternal health, they do not focus extensively on vertical transmission or perinatal outcomes. Hence, the inquiry into whether COVID-19 can be transmitted from a mother to her foetus remains largely unresolved based on the findings of these studies. Generally, the studies reviewed indicate that there was no indication of vertical transmission of COVID-19 from infected mothers to their neonates, in the majority of the papers [20,21,22,24,25,27,29,30,31,32,33,34,35,36], These results are related to other studies’ results which suggested that there is no conclusive evidence of vertical transmission [11,37,38,39,40,41,42,43,44].

On the other hand, according to our results, we found the possibility of vertical transmission only in two papers, those of Ferrazzi et al. [23] from Italy and Nie et al. [26] from China. However, in Ferrazzi et al.’s study, only three infants tested positive from the total of 42, and Nie et al.’s study indicated only one infected neonate from a total of 28 which were born from COVID-19 infected mothers. These results are similar with studies [45,46,47,48]. Especially, Angelidou et al. showed that of the 255 neonates who were born to mothers with SARS-CoV-2 infection, five neonates (2,2%) had a positive test [44] and Mand et al. showed that 2,1% of newborns confirmed SARS-Cov-2 infection were identified [46]. Vertical SARS-CoV-2 transmission is significantly more likely when maternal COVID-19 is severe [48]. The rate of vertical transmission found 5.3% in the systemic review by Jafari et al. [11].

According to the latest data to reduce the risk of transmission, special care should be taken during vaginal assisted delivery by reducing faecal contamination [49]. Not much needs to be done to prevent vertical transmission, other than promoting vaccination. Also, strict hygiene measures should be taken in the delivery room to avoid contamination from maternal biological fluids and health professionals should be adequately trained [50].

## 5. Conclusion

During pregnancy, there may be a process of vertical transmission of the COVID-19 infection from the mother to the foetus. During this period, the frequency of vertical transmission of the virus from the mother to the foetus has not yet been precisely determined but it is rare. The most important preventive measures are vaccination, hygiene measures in the delivery room, and staff training.
